# The effect of *Clostridium butyricum* on symptoms and fecal microbiota in diarrhea-dominant irritable bowel syndrome: a randomized, double-blind, placebo-controlled trial

**DOI:** 10.1038/s41598-018-21241-z

**Published:** 2018-02-14

**Authors:** Yi-Yuan Sun, Ming Li, Yue-Yue Li, Li-Xiang Li, Wen-Zhe Zhai, Peng Wang, Xiao-Xiao Yang, Xiang Gu, Li-Jin Song, Zhen Li, Xiu-Li Zuo, Yan-Qing Li

**Affiliations:** 1 Department of Gastroenterology, Qilu Hospital, Shandong University, Jinan, 250012 China; 2 Laboratory of Translational Gastroenterology, Qilu Hospital, Shandong University, Jinan, 250012 China

## Abstract

Irritable bowel syndrome (IBS) is a common disorder in gastrointestinal system and impairs the quality of life of the patients. *Clostridium butyricum* (*CB*) is a probiotics that has been used in several gastrointestinal diseases. The efficacy of *CB* in treating IBS is still unknown. This prospective, multi-centre, randomized, double-blind, placebo-controlled trial aimed to assess the efficacy and safety of *CB* in treating diarrhea-predominant IBS (IBS-D) and analyze the fecal microbiota after treatment. Two hundred patients with IBS-D were recruited and were given *CB* or placebo for 4 weeks. End points included change from baseline in IBS symptoms, quality of life, stool consistency and frequency. Compared with placebo, *CB* is effective in improving the overall IBS-D symptoms (−62.12 ± 74.00 vs. −40.74 ± 63.67, *P* = 0.038) as well as quality of life (7.232 ± 14.06 vs. 3.159 ± 11.73, *P* = 0.032) and stool frequency (−1.602 ± 1.416 vs. −1.086 ± 1.644, *P* = 0.035). The responder rates are found higher in *CB* compared with the placebo (44.76% vs. 30.53%, *P* = 0.042). The change in fecal microbiota was analyzed and function pathways of *CB* in treating IBS-D were predicted. In conclusion, *CB* improves overall symptoms, quality of life and stool frequency in IBS-D patients and is considered to be used as a probiotics in treating IBS-D clinically.

## Introduction

Irritable bowel syndrome (IBS) is one of the most common gastrointestinal disorders characterized by abdominal pain or discomfort associated with defecation and change in bowel habit^1^. IBS effects 15–20% of the population especially in industrial nations and may impair social and personal functions and effect the quality of life of the patients^2,3^. IBS is classified into 4 subtypes based on the symptoms and diarrhea-predominant IBS (IBS-D) is more prevalent in a community-based data^4^. The concise etiology and pathophysiology of IBS remain unknown, while some factors involve in this process such as gastrointestinal motility, visceral hypersensitivity, psychosocial factors, immune activation and also intestinal microbiota alteration^5,6^. An increase of Firmicutes-associated taxa, a depletion of Bacteroidetes-related taxa and a significantly lower biodiversity of microbes happen in the intestinal microbiota of IBS patient^7,8^. In this way, the improvement of the composition of the intestinal microbiota becomes to the target of treating IBS.

Probiotics are medications that can supplement the intestinal microbiota and improve microbiota characteristics^9^. Several researches prove that probiotics can promote IBS symptoms and quality of life^10–12^. The effects of probiotics include improvement of mucosal barrier function, promoting visceral hypersensitivity, effect on gastrointestinal motility and regulation of immune responses^13–16^. *Clostridium butyricum* (*CB*) is a butyric acid-producing Gram-positive anaerobe which exists in the intestine of humans and has been clinically used in several diseases such as inflammatory bowel disease (IBD) and antimicrobial-associated diarrhea^17–19^. We propose that the protective function of *CB* may exist in treating IBS-D. In this study, we aimed to assess the efficacy and safety of *CB* in IBS-D patients in a multi-centre, randomized, double-blind, placebo-controlled trial and the potential function of *CB* based on intestinal microbiota.

## Methods

### Participants

Male and female outpatients aged 18–65 years who were diagnosed with IBS-D were recruited in three centers in Shandong Province, China. The aim of setting upper age limit was to minimize the number of patients with age-related organic diseases that can lead to IBS-like symptoms. The diagnosis of IBS-D was according to Rome III criteria. Examinations within 3 months were negative including whole blood count, blood chemistry, stool routine, colonoscopy and barium enema examination. Exclusion criteria included other organic gastrointestinal diseases (inflammatory bowel diseases, celiac disease, gastrointestinal infection, gastrointestinal tumor, lactose intolerance, etc); organic diseases (hepatic, renal or cardiac dysfunction, diabetes mellitus, tumor, etc); long-term use of antipsychotics or systemic corticosteroids; the use of antibiotics, probiotics, laxative and other medications that may influence bowel movement for the 4 weeks prior to the study; the examination of colonoscopy and barium enema or the history of acute gastroenteritis in 2 weeks prior to the study; pregnancy or lactation.

### Study design

This was a prospective, multi-centre, randomized, double-blind, placebo-controlled trial designed to investigate the efficacy and safety of the *CB* in the treatment of IBS-D. The trial took place at Qilu Hospital of Shandong University, Taian City Central Hospital and Linyi People’s Hospital between December 2015 and November 2016. The study was carried out in accordance with the Declaration of Helsinki, registered on clinicaltrials.gov (NCT02614963) and was approved by the ethics committees in Shandong University affiliated Qilu Hospital. The trial design was performed according to the CONSORT statement.

All of the patients who fulfilled the inclusion received informed consent before the study. The patients recorded their basic information, symptoms, IBS symptom severity scale (IBS-SSS)^20^, IBS quality of life (IBS-QOL)^21^ score, stool consistency and frequency from questionnaires and provided a stool sample before divided randomly into *CB* and placebo groups. The *CB* capsules (ATaiNing, Qingdao Eastsea Pharmaceutical Co., Ltd., 420 mg per capsule, 1.5 × 10^7^ colony forming units (CFU)/g) were provided in treatment group. The placebo capsule had a same shape, taste and packaging with the *CB* capsule. The medications were labeled by random numbers based on *CB* or placebo and were allotted to patients randomly. All patients and researchers were blinded for *CB* or placebo during the trial. All of the patients in *CB* and placebo groups took 3 capsules 3 times a day for 4 weeks. At the time of discontinuation, the patients visited the clinic at the end of week 4 and recorded their symptoms, IBS-SSS, IBS-QOL score, stool consistency and frequency and adverse events from questionnaires and provided a stool sample. All stool samples were collected for a further analysis using 16 s rRNA pyrosequencing and metagenome sequencing analysis. Other probiotics and medications that might influence the results of the study were not allowed during the whole trial. All patients and investigators were blinded until the study finish.

### Clinical Outcome Assessments

The primary endpoint of our study was the difference in change of IBS symptoms between the two groups as measured by the IBS-SSS^20^ from baseline to week 4. The IBS-SSS contained 5 questions to assess the IBS symptom from 4 aspects: abdominal pain (degree and frequency), bloating, satisfaction with bowel habit and overall interference with QOL. The IBS-SSS total score ranged from 0 to 500 points with 100 points each question. A higher score indicated a more severe condition. Total IBS-SSS score below 175 points represented mild IBS, 175–300 points represented moderate severity and score above 300 points represented severe IBS. A reduction of ≥50 points of total IBS-SSS score was defined as response to treatment^20^.

Secondary endpoints included the difference in changes of IBS-QOL^21^ scores, Bristol stool scale^22^ and stool frequency from baseline to week 4. The IBS-QOL score ranged from 0 to 100 points and a higher score indicated a better QOL. The score contained 34 questions from 8 aspects: dysphoria, interference with activity, body image, health worry, food avoidance, social reaction, sexual concern and relationship. Stool consistency was assessed by the 7-point Bristol stool scale, a higher score indicating a softer stool. Stool frequency was defined as stools per day.

### Stool sample storage and DNA extraction

Stool samples were collected from IBS-D patients in *CB* and placebo groups before (baseline) and after (week 4) treatment. All of the stool samples were immediately stored at −80 °C and were transferred to Majorbio (Shanghai, China) where the total DNA was extracted and tested according to the standardized protocol^23^ for further analysis.

### 16 s rRNA pyrosequencing and metagenome sequencing analysis

16 s rRNA pyrosequencing was processed at Majorbio (Shanghai, China) by using Illumina Miseq system. Similar sequences were clustered into the same operational taxonomy unit (OTU) with a 97% sequence identity. The Chao index, Sobs index and Shannon index were calculated to assess the alpha-diversity in each sample. The cluster analysis based on the Euclidean distance was conducted based on the relative abundances of all OTUs. The principal co-ordinates analysis (PCoA) based on the Braycurtis distance was performed to assess the beta-diversity.

The metagenome sequencing was performed and analyzed at Majorbio (Shanghai, China) by using Illumina Hiseq system. The raw sequences were decoded, denoised, trimmed and then assembled for gene prediction by MetaGene (http://metagene.cb.k.u-tokyo.ac.jp/). The predicted genes were clustered (95% identity, 90% coverage) by CD-HIT (http://www.bioinformatics.org/cd-hit/) into a non-redundant gene catalog. EggNOG (evolutionary genealogy of genes: Non-supervised Orthologous Groups, http://eggnog.embl.de/) and KEGG (Kyoto Encyclopedia of Genes and Genomes, http://www.genome.jp/kegg/) databases were used to predict gene function. The LEfSe (Linear discriminant analysis Effect Size) was conducted to indicate the significant factors between the groups. The STAMP analysis was used to assess function richness between groups. The whole metabolic pathways were mapped by iPath2.0 (http://pathways.embl.de).

### Statistical analysis

The sample size calculation was based on the response rate defined as a reduction of ≥50 points of total IBS-SSS score (35% in the placebo group, 60% in the *CB* group). The size of the sample was 79 per group with the power of 90%, α = 5%. The sample size was also calculated by the change of IBS-SSS score with the power of 80% and α = 5% and the result was smaller than prior calculation. As a result, we planned to enroll a total of 190 patients to allow for a 20% of drop-out.

Clinical data analyses were performed by GraphPad Prism 5 (GraphPad Software, Inc., San Diego, CA) and R 3.1.1 was used in microbiota analysis. The clinical data was analyzed by one researcher (Yi-Yuan Sun) and the analysis of sequencing data was performed by four researchers (Yi-Yuan Sun, Li-Xiang Li, Zhen Li and Ming Li).Missing data were not imputed in the end points. For responder rate, patients with missing data in IBS symptoms were considered to be non-responders. An intention-to-treat analysis was used in responder rate calculation and per-protocol analysis in symptom analysis. The chi-squared analysis was used in proportion data. Symptom differences between groups were tested by nonparametric Wilcoxon test. Data with normal distribution was determined by the Student’s t-test. For microbiota analysis, Wilcoxon test was used in diversity index calculation. Fisher’s exact test was performed in cluster analysis. Different OTUs and PCoA analysis between groups was determined by Kruskal-Wallis test. Gene function differences were tested by ANOSIM analysis. Welch’s t-test was used in STAMP analysis. All P-values were two-sided and were considered as statistical significance below 0.05.

## Results

### Subjects and baseline characteristics

**Figure 1:**
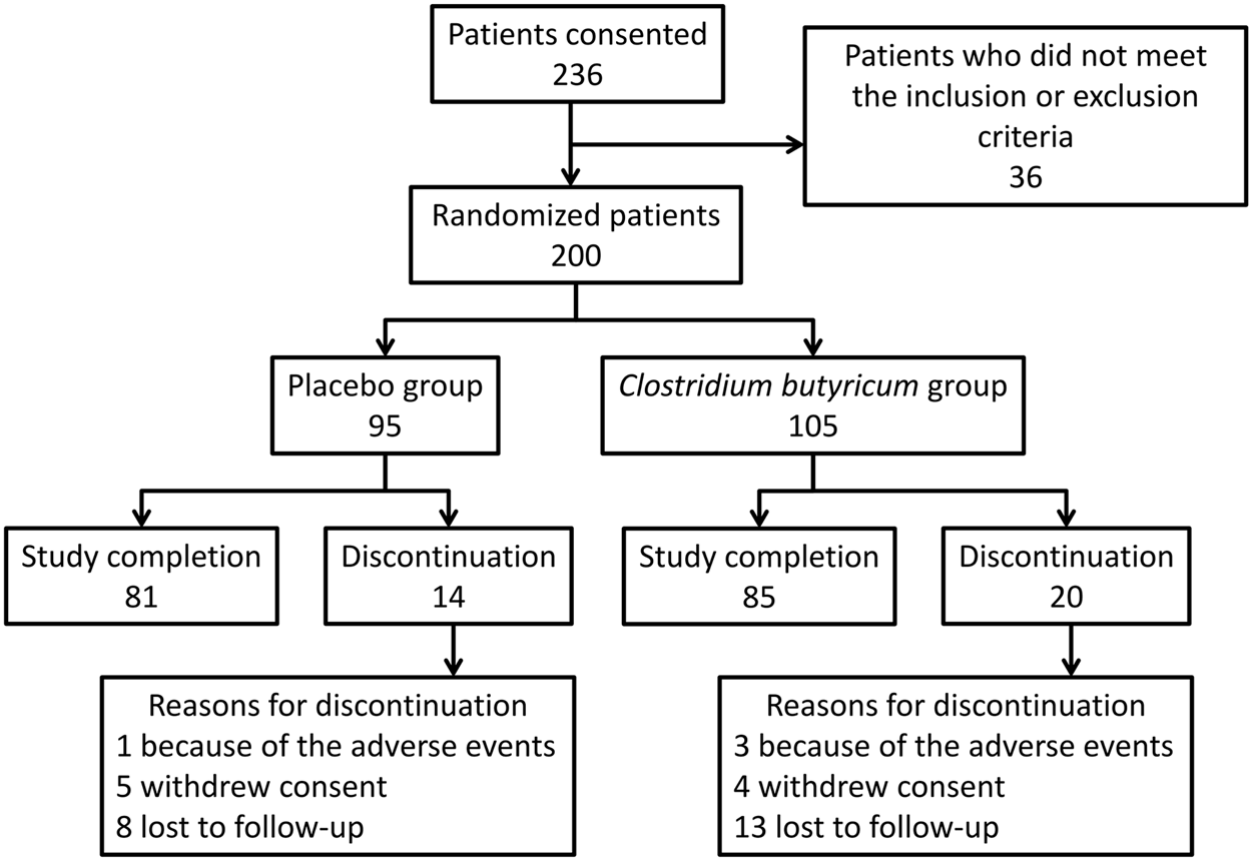
Flow chart of this study. Reasons for discontinuation are shown.

A total of 236 patients were screened in the study and 200 patients met inclusion criteria and were randomized to receive either placebo (N = 95) or *CB* (N = 105). Thirty-four (17.0%) patients did not complete the study: 4 (1 in the placebo group, 3 in the *CB* group) because of the adverse events, 9 (5 in the placebo group, 4 in the *CB* group) who withdrew consent and 21 (8 in the placebo group, 13 in the *CB* group) who were lost to follow-up. A total of 166 patients finished the study, with 81 in placebo group and 85 in *CB* group, respectively (Fig. 1).

In terms of baseline characteristics, demographics were well balanced between the placebo group and the *CB* group. IBS-SSS (232.4 ± 66.09 vs. 243.8 ± 87.93) and IBS-QOL scores (82.66 ± 16.36 vs. 78.90 ± 19.47) showed no significant differences between the placebo group and the *CB* group in baseline (Table 1).

**Table 1:** Demographics and Baseline Characteristics of the patients.

	Placebo group (n = 95)	*Clostridium butyricum* group (n = 105)
Age [mean (SD)]	44.91 (13.01)	43.00 (12.45)
Sex		
Female (%)	42 (44.21%)	42 (40.00%)
Male (%)	53 (55.79%)	63 (60.00%)
Baseline IBS-SSS [mean (SD)]	232.4 (66.09)	243.8 (87.93)
Baseline IBS-QOL score [mean (SD)]	82.66 (16.36)	78.90 (19.47)

### Assessment of IBS symptoms

**Figure 2:**
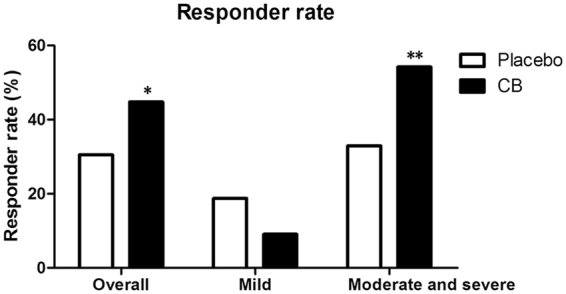
Responder rate after treatment in placebo and *Clostridium butyricum* (*CB*) groups. The overall responder rate was higher in the *CB* group compared with that in the placebo group (*P* = 0.042). The responder rate of patients with moderate to severe symptoms in the *CB* group was significantly higher than that in the placebo group (*P* = 0.007). *P < 0.05, **P < 0.01.

The primary endpoint was the reduction of IBS-SSS from baseline to week 4. There was a significant reduction of IBS-SSS from baseline to week 4 in the *CB* group compared with that in the placebo group (−62.12 ± 74.00 vs. −40.74 ± 63.67, *P* = 0.038) (Table 2). This change in IBS-SSS indicated the improvement of IBS symptoms in the *CB* group compared with the placebo group. The changes in individual component scores for IBS-SSS were also analyzed. There was a significant reduction in the component scores for bowel habit (−20.71 ± 22.10 vs. −12.84 ± 21.48, *P* = 0.014) and QOL satisfaction (−13.18 ± 19.41 vs. −6.667 ± 13.78, *P* = 0.018) in the *CB* group compared with that in the placebo group. However, there were no significant differences in the component scores for pain (−22.59 ± 36.62 vs. −16.54 ± 37.12, *P* = 0.276) and bloating (−5.647 ± 19.18 vs. −4.691 ± 16.21, *P* = 0.485) between patients taking *CB* and placebo. The responder rate was assessed by a reduction of IBS-SSS ≥50 points. A higher overall responder rate was found in the *CB* group compared with that in the placebo group (44.76% vs. 30.53%, *P* = 0.042) by an ITT analysis (Fig. 2). For the patients with moderate to severe symptoms (IBS-SSS >175 points), the responder rate in the *CB* group was significantly higher than that in the placebo group (54.22% vs. 32.91%, *P* = 0.007). The result showed a significant efficacy of *CB* in treating IBS-D especially for the patients with moderate to severe symptoms.

**Table 2:** IBS-SSS scores: baseline to week 4.

	IBS-SSS [Mean (SD)] and change from baseline [Mean (SD)]						
	Baseline		Week 4		Change from baseline		
	Placebo	CB	Placebo	CB	Placebo	CB	P value
IBS-SSS total	230.4 (67.16)	228.5 (83.63)	189.6 (70.36)	166.4 (66.56)	−40.74 (63.67)	−62.12 (74.00)	0.038
Pain	95.31 (37.98)	92.00 (41.31)	78.77 (37.86)	69.41 (35.13)	−16.54 (37.12)	−22.59 (36.62)	0.276
Bloating	35.31 (13.57)	35.53 (19.35)	30.62 (14.17)	29.88 (15.62)	−4.691 (16.21)	−5.647 (19.18)	0.485
Bowel habit	55.56 (19.49)	53.88 (20.94)	42.73 (19.43)	33.18 (18.52)	−12.84 (21.48)	−20.71 (22.10)	0.014
QOL satisfaction	44.20 (16.95)	47.06 (19.93)	37.53 (15.29)	33.88 (16.04)	−6.667 (13.78)	−13.18 (19.41)	0.018

### Secondary endpoints

Secondary endpoints included the changes of IBS-QOL scores, Bristol stool scale and stool frequency from baseline to week 4. Significant improvement in change of overall IBS-QOL score was observed in the *CB* group compared with the placebo group (7.232 ± 14.06 vs. 3.159 ± 11.73, *P* = 0.032) (Table 3). Furthermore, there was a significant improvement in change of interference with activity (8.866 ± 15.81 vs. 2.822 ± 13.34, *P* = 0.003) and health worry (12.25 ± 18.03 vs. 2.469 ± 16.43, *P* < 0.001), but not dysphoria, body image, food avoidance, social reaction, sexual concerns, relationship (8.934 ± 17.67 vs. 5.980 ± 16.22, *P* = 0.135; 3.603 ± 14.12 vs. 0.7716 ± 14.02, *P* = 0.072; 7.451 ± 22.49 vs. 4.630 ± 20.28, *P* = 0.381; 4.853 ± 16.38 vs. 0.6173 ± 10.44, *P* = 0.074; 5.147 ± 17.07 vs. 1.080 ± 13.29, *P* = 0.119; 3.039 ± 13.96 vs. 3.601 ± 13.88, *P* = 0.751, respectively) in the *CB* group compared with the placebo group.

**Table 3:** IBS-QOL scores: baseline to week 4.

	IBS-QOL [Mean (SD)] and change from baseline [Mean (SD)]						
	Baseline		Week 4		Change from baseline		
	Placebo	CB	Placebo	CB	Placebo	CB	P value
QOL overall	82.88 (16.14)	81.28 (18.85)	86.04 (13.74)	88.51 (14.40)	3.159 (11.73)	7.232 (14.06)	0.032
Dysphoria	77.85 (23.08)	79.34 (23.88)	83.83 (18.64)	88.27 (19.06)	5.980 (16.22)	8.934 (17.67)	0.135
Activity interference	85.14 (17.83)	80.71 (20.86)	87.96 (13.81)	89.58 (14.86)	2.822 (13.34)	8.866 (15.81)	0.003
Body image	91.20 (15.99)	90.00 (16.49)	91.98 (13.08)	93.60 (11.33)	0.7716 (14.02)	3.603 (14.12)	0.072
Health worry	77.26 (20.83)	72.16 (23.34)	79.73 (20.49)	84.41 (20.32)	2.469 (16.43)	12.25 (18.03)	0.000
Food avoidance	68.31 (22.65)	65.39 (22.14)	72.94 (21.02)	72.84 (19.89)	4.630 (20.28)	7.451 (22.49)	0.381
Social reaction	87.35 (17.76)	86.84 (20.00)	87.96 (17.22)	91.69 (14.61)	0.6173 (10.44)	4.853 (16.38)	0.074
Sexual	90.28 (21.01)	86.62 (24.15)	91.36 (18.50)	91.76 (15.97)	1.080 (13.29)	5.147 (17.07)	0.119
Relationship	89.20 (19.38)	90.20 (16.82)	92.80 (15.91)	93.24 (12.76)	3.601 (13.88)	3.039 (13.96)	0.751

**Figure 3:**
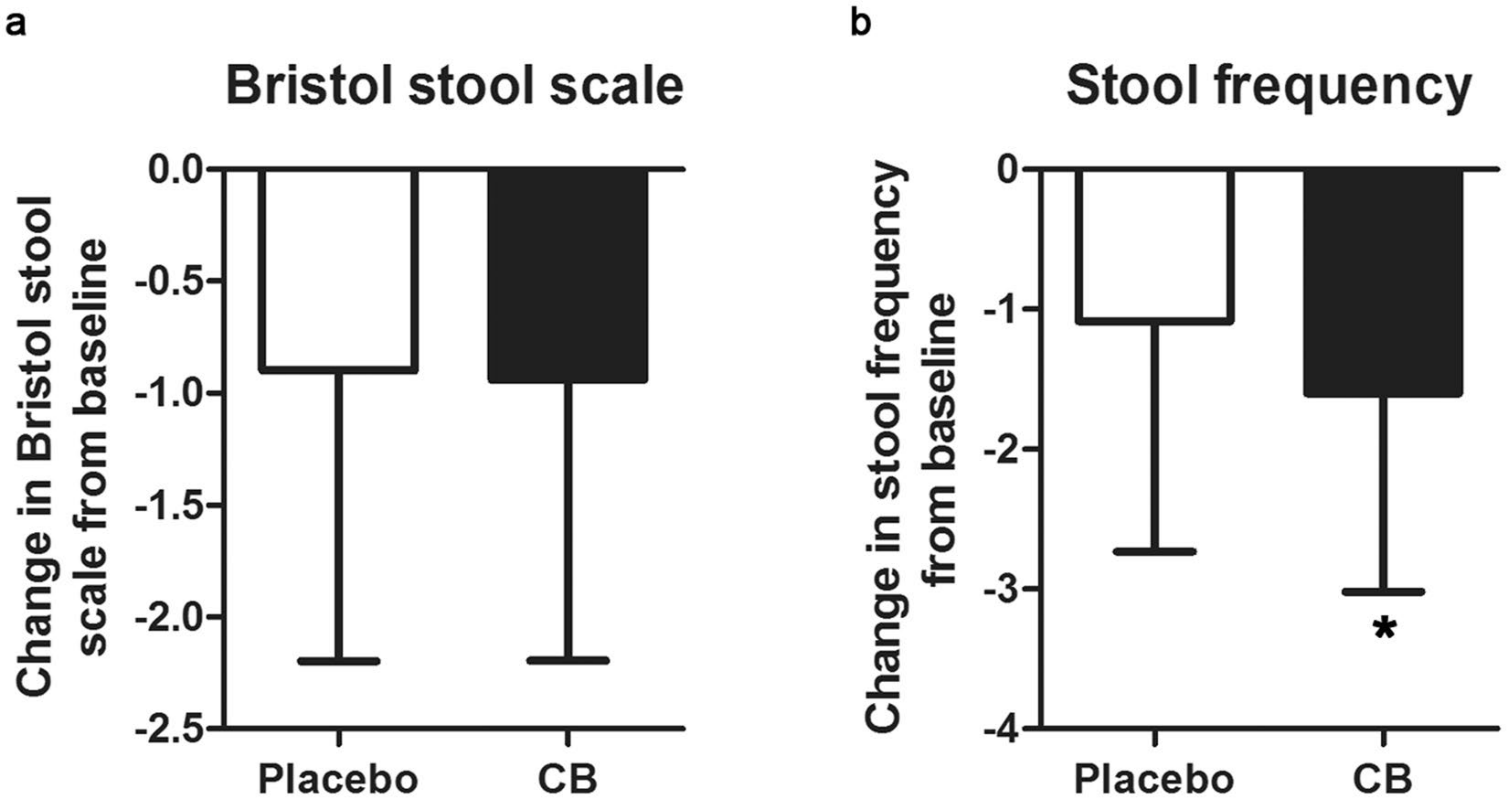
The improvement of stool consistency and frequency in placebo and *Clostridium butyricum* (*CB*) groups. (**a**) No significant difference was found in change of Bristol stool scale from baseline to week 4 between the *CB* group and the placebo group (*P* = 0.259). (**b**) The reduction of stool frequency in *CB* group was significantly superior than that in the placebo group (*P* = 0.035). *P < 0.05.

A total of 164 patients (80 in the placebo group, 84 in the *CB* group) assessed Bristol stool scale and 124 patients (58 in the placebo group, 66 in the *CB* group) assessed stool frequency (stools per day) at baseline and week 4 (Fig. 3). No significant difference was found in change of Bristol stool scale from baseline to week 4 between the *CB* group and the placebo group (−1.012 ± 1.078 vs. −0.9000 ± 1.296, *P* = 0.259). However, the improvement of stool frequency from baseline to week 4 was significantly superior in the *CB* group than in the placebo group (−1.602 ± 1.416 vs. −1.086 ± 1.644, *P* = 0.035).

### Safety

The patients took medications as we had informed. Only 8 adverse events were reported in this study, 2 (2.11%) in the placebo group and 6 (5.71%) in the *CB* group. Among these adverse events, 6 were worse abdominal pain (2 in the placebo group, 4 in the *CB* group), 1 was bloating in the *CB* group and 1 was hyperactive bowel sound in the *CB* group. No severe adverse events have been recorded in either group.

### 16 s rRNA pyrosequencing analysis of stool samples

A total of 200 stool samples from baseline and week 4 of 100 patients (42 in the placebo group, 58 in the *CB* group) were analyzed in this study using 16 s rRNA pyrosequencing. The demographics of the placebo group and the *CB* group were shown in Supplementary Table 1. No significant differences were found between two groups.

**Figure 4:**
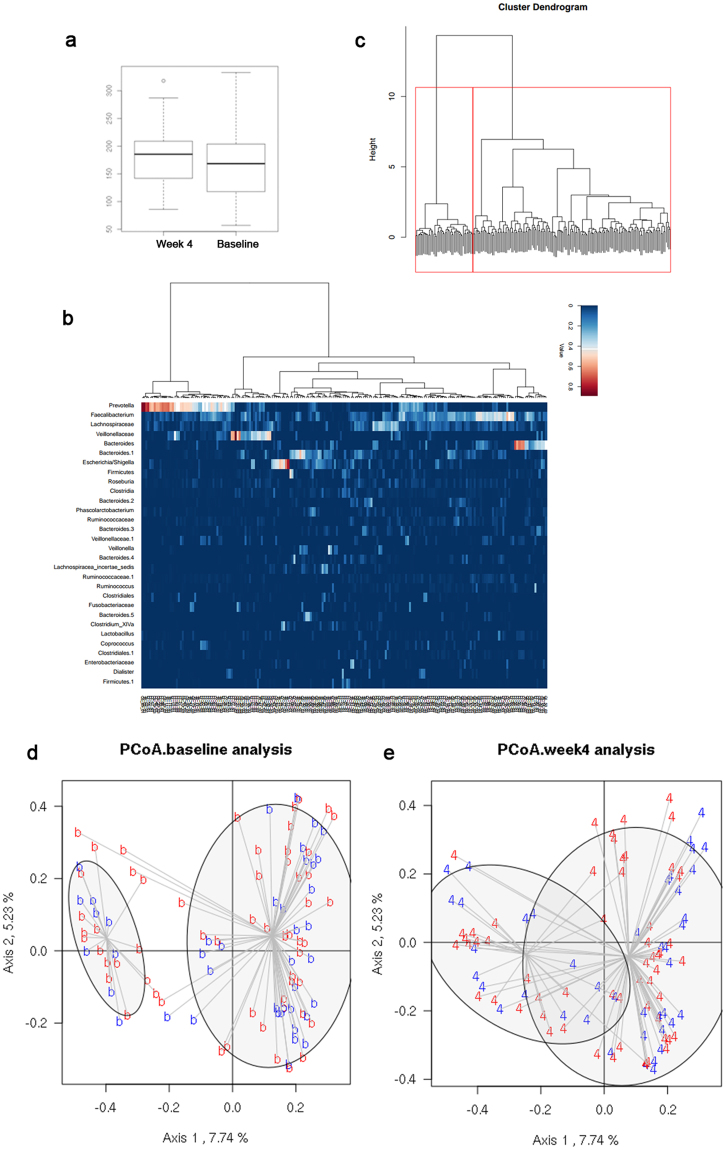
16 s rRNA pyrosequencing analysis of stool samples. (**a**) Wilcoxon test of Sob index showed an increased tendency after treating with *Clostridium butyricum* (*CB*) (*P* = 0.063). (**b**) Heat-map plot with 30 most abundant OTUs in all stool samples. (**c**) Two clusters were observed using Fisher’s exact test. (**d**,**e**) The PCoA plot of placebo and *CB* groups in baseline and week 4.

A total of 7,406,811 sequences were finally analyzed and were clustered into 797 OTUs. We compared microbial diversity between baseline and week 4 levels with Chao, Sobs and Shannon index. No significant differences were found in both two groups. Sobs index showed an increased tendency after treating with *CB* (*P* = 0.063), indicating a potential function of *CB* in increasing microbial community richness (Fig. 4a).

To evaluate the whole microbial community in all stool samples, we plotted the 30 most abundant OTUs in a heat-map plot (Fig. 4b). *Prevotella*, *Faecalibacterium*, *Lachnospiraceae*, *Veillonellaceae* and *Bacteroides* were the most abundant OTUs in most stool samples. A cluster analysis was performed to determine whether the microbiota was clustered and two clusters were found based on the abundance of all of the variable taxa (Fig. 4c). There were no significant differences between two clusters in both baseline (*P* = 0.644) and week 4 (*P* = 0.805) level. We then evaluated the microbial community in placebo group and *CB* group at baseline and week 4. Parsimony test showed a significant difference between two groups at week 4 (*P* = 0.016). To confirm this result, a PcoA analysis was performed (Fig. 4d,e). The microbial community was similar in two groups at baseline but a significant difference was found at week 4 (χ^2^ = 7.006, df = 1, *P* = 0.008).

We further analyzed the microbiota differences between placebo group and *CB* group in OTU level. 187 and 137 discriminating OTUS were found between two groups at baseline and week 4 respectively. 45 significantly changed OTUs from baseline to week 4 were found between two groups (Supplementary Table 2). These OTUs might indicate the change of intestinal microbiota during the treatment of *CB*. *Clostridium sensu stricto* was one of the most significantly changed OTU between two groups. No significance was observed at baseline between placebo group and *CB* group (0.0006118 ± 0.001396 vs. 0.001860 ± 0.004609, *P* = 0.572) but a significant reduction of *Clostridium sensu stricto* was existed in *CB* group (−0.0007532 ± 0.005059 vs. 0.01950 ± 0.007487, *P* = 0.023). We then analyzed different OTUs between responder and non-responder in *CB* group. There was a tendency of more *Clostridium sensu stricto* in responder at baseline (0.003260 ± 0.005983 vs. 0.0007221 ± 0.002685, *P* = 0.057) and a significant reduction of *Clostridium sensu stricto* after treating with *CB* in responder compared with non-responder (−0.002445 ± 0.006260 vs. 0.0006213 ± 0.003333, *P* < 0.001).

### Function prediction of *CB* in treating IBS-D by metagenomic analysis

Fifty-two stool samples from baseline and week 4 of 26 patients (13 per group) were analyzed via metagenome sequencing. The samples of two groups were well paired. The demographics of the placebo group and the *CB* group were shown in Supplementary Table 3 and no significant differences were found between two groups.

**Figure 5:**
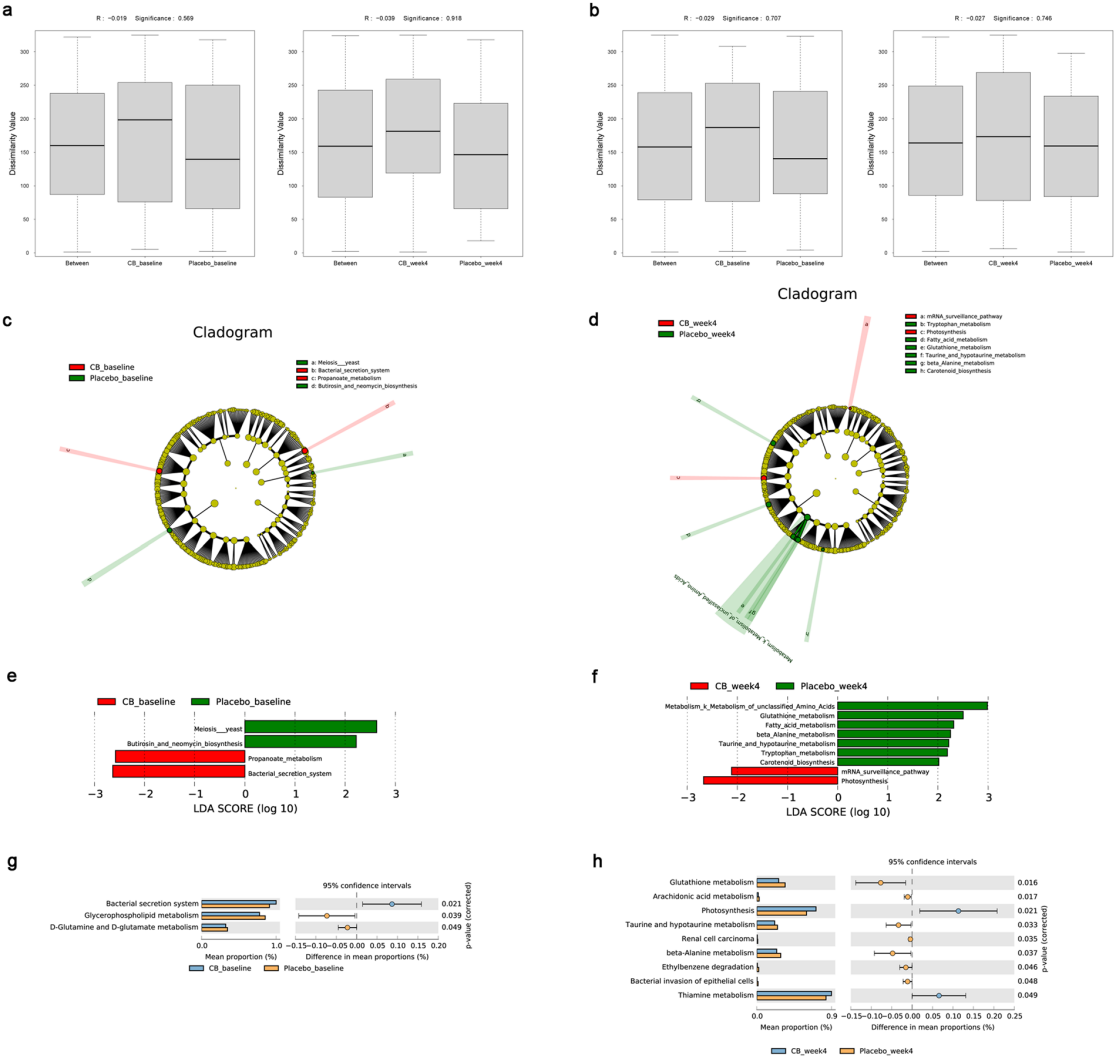
Metagenomic analysis of stool samples. (**a**) ANOSIM analysis of eggNOG between placebo and *Clostridium butyricum* (*CB*) groups at baseline and week 4. (**b**) ANOSIM analysis of KEGG between placebo and *CB* groups at baseline and week 4. No significant differences were observed between two groups in baseline and week 4 for eggNOG (*P* = 0.569 in baseline, *P* = 0.918 in week 4) and KEGG (*P* = 0.707 in baseline, *P* = 0.746 in week 4) analysis. (**c**,**d**) LEfSe analysis of KEGG pathways between placebo and *CB* groups at baseline and week 4. (**e**,**f**) LDA score of LEfSe analysis at baseline and week 4. (**g**,**h**) STAMP analysis of KEGG pathways between placebo and *CB* groups at baseline and week 4.

A total of 13,716,343 genes were tested and finally 2,696,668 genes were cataloged into a non-redundant gene catalog for further analysis. The eggNOG and KEGG annotation were used for function prediction of *CB*. We identified 17,454 eggNOG orthologues and 5,971 KEGG orthologues totally. However, no significant differences were observed between placebo group and *CB* groups in baseline and week 4 for eggNOG (*P* = 0.569 in baseline, *P* = 0.918 in week 4) and KEGG (*P* = 0.707 in baseline, *P* = 0.746 in week 4) analysis (Fig. 5a,b).

**Figure 6:**
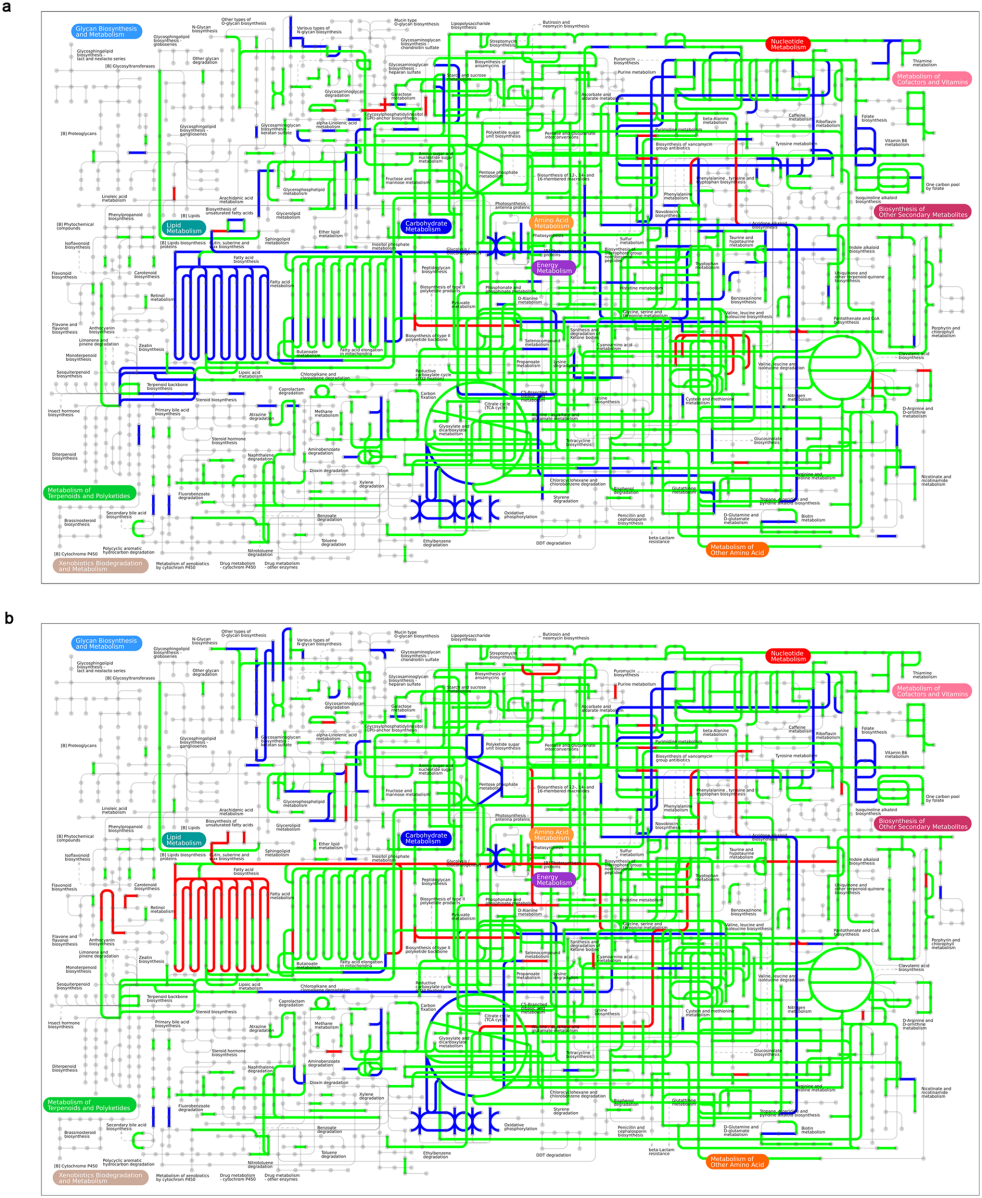
The whole metabolic pathways of *Clostridium butyricum* (*CB*) and placebo via iPath. The blue lines were metabolic pathways dominant in *CB* group. The red lines were metabolic pathways dominant in placebo group. The green lines were co-pathways in both groups. (**a**) The metabolic pathways of two groups at baseline. (**b**) The metabolic pathways of two groups at week 4.

Further we focused on the specific eggNOG orthologues and KEGG pathways between placebo and *CB* groups. No significant differences were shown between two groups. However, several KEGG pathways were included to distinguish two groups after treatment using the LEfSe method (Fig. 5c–f). The *CB* group was associated with mRNA surveillance pathway and photosynthesis and the placebo group was more correlated with pathways such as amino acid metabolism, glutathione metabolism, fatty acid metabolism, beta-alanine metabolism, taurine and hypotaurine metabolism, tryptophan metabolism and carotenoid biosynthesis. Function richness was assessed by STAMP analysis (Fig. 5g,h). The *CB* group was enriched in photosynthesis and thiamine metabolism; however the placebo group was enriched in pathways including glutathione metabolism, arachidonic acid metabolism, taurine and hypotaurine metabolism, beta-alanine metabolism, bacterial invasion of epithelial cells, etc. The whole metabolic pathways of *CB* and placebo were shown in Fig. 6. All above analysis may demonstrate the potential mechanisms of *CB* in treating IBS-D.

## Discussion

This prospective, multi-centre, randomized, double-blind, placebo-controlled study indicates that *CB* has advantages in treating IBS-D. In this study, *CB* promotes the overall IBS symptoms based on bowel habit and quality of life compared with placebo. Interference with activity, health worry and stool frequency are main improvements in bowel habit and quality of life. The responder rates are found higher in *CB* group compared with the placebo group especially in moderate and severe patients. In the fecal microbiota analysis, we demonstrate no significant differences in microbial diversity before and after treatment between *CB* and placebo groups. The microbial community is similar in two groups at baseline but a significant difference is found at week 4. The different OTUs are analyzed and *Clostridium sensu stricto* is one of the most significantly changed OTU between two groups. Further analysis shows a significant reduction of *Clostridium sensu stricto* in responder compared with non-responder after treating with *CB*. Function prediction of *CB* is assessed by metagenomic analysis. Several pathways such as amino acid metabolism, fatty acid metabolism and tryptophan metabolism may play an essential role in treating IBS-D.

Several studies have researched on the effects of probiotics on IBS^3,11,12^. While some studies show a benefit in IBS symptoms with probiotics, others report limited benefits^24^. A recent meta-analyse suggested that probiotic therapy improves the overall symptoms and quality of life, but not individual IBS symptoms such as abdominal pain and bloating^10^. In this study, we demonstrate a similar result. Probiotic *CB* improves overall IBS symptoms in IBS-D patients compared with placebo. Further analysis shows that the improvement in IBS symptom is related to the change in quality of life and bowel habit, but not the abdominal pain and bloating. The overall responder rate (44.76%) of *CB* is higher than that of placebo but lower than that in some other studies^2^. We explain that a higher drop-out rate (19.05%) and a limitation in the reduction of IBS-SSS ≥50 points in mild IBS patients lead to this result, and also this is why the responder rate in moderate to severe patients is higher (54.22%).

The *CB* has been clinically used for several years but the effect and mechanism of the *CB* on IBS is unclear. Asuka Yasueda *et al*.^18^ have found that *CB* has benefit in the prevention of pouchitis and analyzed the characteristic intestinal flora after treating with *CB* or placebo. In an experimental colitis model in mice, *CB* suppresses symptoms via immune pathways^25,26^ but does not alter the composition of gut microbiota^26^. Since IBS is different from the organic gastrointestinal diseases, we focus on the change of microbiota during the use of *CB*. However, no significant differences are found in week and change of Bristol stool scale in both clinic symptoms and fecal samples and we suppose that this difference in baseline is permitted. As a result, the diversity analysis shows no differences before and after treatment in two groups but the microbial communities are different after treating with *CB* or placebo. A typical genus, *Clostridium sensu stricto*, is confirmed as a potential factor of *CB* in IBS-D treatment.

Several studies have shown an increase of short-chain fatty acids (SCFAs) level in IBS^27,28^. SCFAs, such as butyrate and acetate, have many benefits and play an important role in intestine function: enhancing the intestinal barrier, improving intestinal microbiota, regulating immune system and promoting gastrointestinal motility^29–32^. However, butyrate may promote visceral hypersensitivity^33,34^, which is a pathophysiological mechanism in IBS. *Clostridium sensu stricto* is a representative cluster of the genus *Clostridium* and exhibits SCFAs^35^ and it is reported that there is an increase of *Clostridium* in fecal samples of IBS patients^36,37^. In this study, we find a decrease of *Clostridium sensu stricto* after treating with *CB* and also a significant reduction of *Clostridium sensu stricto* in responder compared with non-responder. It is supposed to be a potential mechanism in treating IBS-D by *CB*.

In this study, we demonstrate several significantly different pathways between the placebo group and the *CB* group. Compared with placebo, treating with *CB* may downregulate pathways including fatty acid metabolism, beta-alanine metabolism, tryptophan metabolism, etc. Increased level of SCFAs is observed in IBS^27,28^. In the present study, we declare a decrease of SCFA-produced *Clostridium sensu stricto* after treating with *CB*, which may improve fatty acid metabolism in IBS patients. Increased level of alanine is reported in stool samples of IBS patients^38^ and the metabolite of tryptophan, serotonin, play an important role in gastrointestinal motility and IBS^39,40^. The improvement of alanine and tryptophan metabolism may reflect the alanine and serotonin in bowel and ameliorate the IBS symptoms.

In general, the analysis of stool samples between the placebo group and the *CB* group is not as significant as we expected. Associated with the clinical symptoms, the improvement of IBS symptom is based on quality of life and stool frequency, but not abdominal pain and bloating. These results may indicate a limited change in bowel microbiota or regulated pathways.

There are several limitations of this study. We analyze the IBS symptoms and fecal microbiota only at baseline and week 4 without a long-term surveillance. The continuous change in IBS symptom scores and fecal microbiota and the reaction after discontinuation cannot be proved. Furthermore, as only one dose of *CB* is used in this study, we cannot confirm the efficacy of *CB* with different doses in IBS treatment and the optimal dosing regimen. A further study of clinic use of *CB* is still needed. Another limitation is that lack of diet evaluation. However, we consider that a large sample size and randomization may reduce the difference of diet among patients and the effect of diet on the final results is weak. We focus on the fecal microbiota as a mechanism of *CB* in treating IBS-D. Since it is reported that *CB* participates in intestine function with some other mechanisms, such as immune regulation, a more detailed mechanism of *CB* in basic medicine and clinic is required.

In conclusion, this study has shown that *CB* is effective in improving the overall IBS-D symptoms, especially in bowel habit and quality of life. The responder rates are found higher in *CB* compared with the placebo. The fecal microbiota analysis shows a different microbial community after treating with *CB* and a typical genus, *Clostridium sensu stricto*, is decreased with the treatment of *CB*. *CB* is involved in several metabolic pathways to perform its function in treating IBS-D.

## Electronic supplementary material

Supplementary Table 1–3
