# Supplementary material for: The effect of Clostridium butyricum on symptoms and fecal microbiota in diarrhea-dominant irritable bowel syndrome: a randomized, double-blind, placebo-controlled trial
Source: Sci Rep. 2018 Feb 14;8:2964. doi: 10.1038/s41598-018-21241-z (PMC5813237; doi:10.1038/s41598-018-21241-z)
Supplement: Supplementary file 1 — Supplementary Table 1–3 [file 41598_2018_21241_MOESM1_ESM.doc]

**The effect of *Clostridium butyricum* on symptoms and fecal microbiota in diarrhea-dominant irritable bowel syndrome: a randomized, double-blind, placebo-controlled trial**

Yi-Yuan Sun^1,2^, Ming Li^1,2^, Yue-Yue Li^1,2^, Li-Xiang Li^1,2^, Wen-Zhe Zhai^1,2^, Peng Wang^1,2^, Xiao-Xiao Yang^1,2^, Xiang Gu^1,2^, Li-Jin Song^1,2^, Zhen Li^1,2^, Xiu-Li Zuo^1,2^, Yan-Qing Li^1,2*^

^1^Department of Gastroenterology, Qilu Hospital, Shandong University, Jinan, 250012, China.

^2^Laboratory of Translational Gastroenterology, Qilu Hospital, Shandong University, Jinan, 250012, China

* Correspondence to: Professor Yan-Qing Li, Department of Gastroenterology, Qilu Hospital, Shandong University, No. 107, Wenhua Xi Road, Jinan, 250012, China.

Fax: +86-531-82166090

Telephone: +86-531- 82166090

E-mail: liyanqing@sdu.edu.cn

**Supplementary Table 1: Demographics and Baseline Characteristics of the patients with stool samples for 16s rDNA pyrosequencing analysis**

|  | **Placebo group**  **(n=42)** | ***Clostridium butyricum* group (n=58)** |
| --- | --- | --- |
| **Age [mean (SD)]** | 49.76 (12.29) | 44.72 (12.50) |
| **Sex** |  |  |
| **Female (%)** | 15 (35.71) | 23 (39.66) |
| **Male (%)** | 27 (64.29) | 35 (60.34) |
| **Baseline IBS-SSS [mean (SD)]** | 229.5 (74.99) | 232.1 (94.33) |
| **Baseline IBS-QOL score**  **[mean (SD)]** | 83.28 (17.18) | 78.98 (21.72) |
| **Baseline Bristol stool scale**  **[mean (SD)]** | 5.929 (0.8874) | 6.216 (0.8691) |

**Supplementary Table 2: The change of OTUs from baseline to week 4 between placebo and *Clostridium butyricum*** groups

| **OTU number** | **Taxon** | **P value** |
| --- | --- | --- |
| **28** | *Enterobacteriaceae* | 0.0164 |
| **63** | *Clostridia* | 0.0393 |
| **78** | *Parabacteroides* | 0.0169 |
| **81** | ***Clostridium_sensu_stricto*** | **0.0230** |
| **110** | *Streptococcus* | 0.0042 |
| **118** | *Alistipes* | 0.0372 |
| **134** | *Clostridiales* | 0.0263 |
| **142** | *Veillonella* | 0.0439 |
| **144** | *Firmicutes* | 0.0226 |
| **145** | *Clostridiales* | 0.0233 |
| **150** | *Clostridiales* | 0.0237 |
| **153** | *Bacteroides* | 0.0392 |
| **159** | *Clostridiales* | 0.0382 |
| **161** | *Clostridiales* | 0.0084 |
| **189** | *Clostridia* | 0.0245 |
| **191** | *Clostridium_IV* | 0.0207 |
| **199** | *Clostridia* | 0.0007 |
| **209** | *Negativicutes* | 0.0030 |
| **235** | *Alistipes* | 0.0284 |
| **245** | *Negativicutes* | 0.0449 |
| **268** | *Erysipelotrichales* | 0.0293 |
| **273** | *Butyricimonas* | 0.0459 |
| **291** | *Betaproteobacteria* | 0.0481 |
| **321** | *Veillonellaceae* | 0.0277 |
| **340** | *Porphyromonadaceae* | 0.0068 |
| **345** | *Bacteria* | 0.0141 |
| **366** | *Saccharibacteria_genera_incertae_sedis* | 0.0038 |
| **388** | *Firmicutes* | 0.0089 |
| **429** | *Clostridiales* | 0.0339 |
| **444** | *Clostridiales* | 0.0424 |
| **454** | *Bacteria* | 0.0499 |
| **465** | *Clostridia* | 0.0139 |
| **474** | *Firmicutes* | 0.0449 |
| **476** | *Firmicutes* | 0.0076 |
| **524** | *Bacteria* | 0.0292 |
| **594** | *Pasteurellaceae* | 0.0314 |
| **598** | *Clostridia* | 0.0449 |
| **616** | *Rikenellaceae* | 0.0042 |
| **631** | *Comamonadaceae* | 0.0017 |
| **645** | *Bacteria* | 0.0298 |
| **660** | *Corynebacterium* | 0.0149 |
| **684** | *Bacteria* | 0.0063 |
| **701** | *Bacteria* | 0.0262 |
| **710** | *Erysipelotrichaceae_incertae_sedis* | 0.0409 |
| **761** | *Bacteria* | 0.0197 |

**Supplementary Table 3**: Demographics and Baseline Characteristics of the patients with stool samples for metagenomic analysis

|  | **Placebo group**  **(n=42)** | ***Clostridium butyricum* group**  **(n=58)** |
| --- | --- | --- |
| **Age [mean (SD)]** | 43.31 (12.00) | 42.54 (11.78) |
| **Sex** |  |  |
| **Female (%)** | 7 (53.85) | 7 (53.85) |
| **Male (%)** | 6 (46.15) | 6 (46.15) |
| **Baseline IBS-SSS [mean (SD)]** | 233.8 (51.24) | 226.2 (60.21) |
| **Baseline IBS-QOL score**  **[mean (SD)]** | 86.14 (10.42) | 86.43 (10.87) |
| **Baseline Bristol stool scale [mean (SD)]** | 5.769 (0.6330) | 5.615 (1.175) |
